# Why Was the Policy Idea on the Health Benefits Package Advisory Panel Gazetted in Kenya? A Retrospective Policy Analysis

**DOI:** 10.34172/ijhpm.7608

**Published:** 2024-07-08

**Authors:** Rahab Mbau, Anna Vassall, Lucy Gilson, Edwine Barasa

**Affiliations:** ^1^Department of Global Health and Development, London School of Hygiene and Tropical Medicine, London, UK.; ^2^Health Economics Research Unit, KEMRI Wellcome Trust Research Programme, Nairobi, Kenya.; ^3^School of Public Health and Family Medicine, University of Cape Town, Cape Town, South Africa.; ^4^Centre for Global Health and Tropical Medicine, Nuffield Department of Medicine, University of Oxford, Oxford, UK.; ^5^Institute of Healthcare Management, Strathmore University, Nairobi, Kenya.

**Keywords:** Policy Analysis, Multiple Streams Theory, Procedural Policy, Kenya

## Abstract

**Background:**

In 2018, Kenya’s Ministry of Health (MoH) gazetted the Health Benefits Package Advisory Panel (HBPAP) to develop a benefits package for its universal health coverage (UHC) programme. In this study, we examine the political process that led to the gazettement of the HBPAP.

**Methods:**

We conducted a case study based on semi-structured interviews with 20 national-level participants and, reviews of documents such as organizational and media reports. We analyzed data from the interviews and documents thematically using the Braun and Clarke’s six step approach. We identified codes and themes deductively using Kingdon’s Multiple Streams Theory which postulates that the successful emergence of a policy follows coupling of three streams: the problem, policy, and politics streams.

**Results:**

We found that the problem stream was characterized by fragmented and implicit healthcare priority-setting processes that led to unaffordable, unsustainable, and wasteful benefits packages. A potential policy solution for these problems was the creation of an independent expert panel that would use an explicit and evidence-based healthcare priority-setting process to develop an affordable and sustainable benefits package. The political stream was characterized by the re-election of the government and the appointment of a new Cabinet Secretary for Health. Coupling of the problem, policy, and political streams occurred during a policy window that was created by the political prioritization of UHC by the newly re-elected government. Policy entrepreneurs who included health economists, health financing experts, health policy analysts, and health systems experts leveraged this policy window to push for the establishment of an independent expert panel as a solution for the issues identified in the problem stream. They employed strategies such as forming networks, framing, marshalling evidence, and utilizing political connections.

**Conclusion:**

Applying Kingdon’s theory in this study was valuable in explaining why the HBPAP policy idea was gazetted. It demonstrated the crucial role of policy entrepreneurs and the strategies they employed to couple the three streams during a favourable policy window. This study contributes to the body of literature on healthcare priority-setting processes with an unusual analysis focused on a key procedural policy for such processes.

## Background

**Key Messages**

**Implications for policy makers**

- Gazettement of a procedural policy such as Health Benefits Package Advisory Panel (HBPAP) is a political process that can be explained using concepts from the Kingdon’s Multiple Streams Theory.
- Technocrats such as health economists, health financing experts, health policy analysts, and health systems experts can play important roles in identifying healthcare priority-setting problems and developing potential solutions to these problems.
- Technocrats can also act as policy entrepreneurs by taking advantage of policy windows to couple the problem, policy, and politics streams. They can employ strategies such as forming networks, framing, marshalling evidence, and utilizing political connections to achieve this.
- Administrative changes, such as presidential elections or appointment of new cabinet secretaries for health, can create windows of opportunities for emergence of new policies.

**Implications for the public**

This study shows that attentive publics such as the national and county government stakeholders can influence the political process of introducing procedural policies by providing their opinions on potential policy ideas that may lead to explicit allocation of resources. This is important because lack of harmonized and clear benefits packages is an indication of poor resource allocation processes that are unclear, fragmented, and not informed by evidence. This undermines equitable access to services across population groups as was the case in Kenya. In addition, members of the public can use their constitutional right to vote to influence legislative changes which in turn shape the climate for policy change within the health sector.

The world has set global targets to achieve universal health coverage (UHC) by 2030 as part of the sustainable development goals.^1^ Under UHC, anyone in need can obtain good-quality essential health services such as health promotion, disease prevention, diagnosis, treatment, rehabilitation, and palliation with no financial difficulties.^2^ The attainment of UHC is constrained by the available resources in every country (irrespective of income level) which are insufficient to meet the cost of providing all effective health services.^3-7^

 In addition to resource scarcity, approximately 20%-40% of health sector spending is wasted through inefficient allocation of resources.^5^ This wastage has partly been attributed to implicit healthcare priority-setting processes which are driven by historical patterns and stakeholder interests.^5,8^ Healthcare priority-setting refers to decision-making regarding allocation of resources across competing uses.^9,10^ Healthcare priority-setting can be implicit where it is unknown how or by whom resource allocation decisions are made or explicit where it is known how and by whom resource allocation decisions are made.^11^ While implicit processes are ad hoc and unsystematic, explicit healthcare priority-setting processes are deliberative, evidence-based, inclusive, and systematic.^11^

 Resource constraints and continued wastage have generated interest in explicit healthcare priority-setting processes to inform UHC-related decisions on covering more people, expanding services, and reducing out-of-pocket payments.^3,11,12^ Introducing an explicit healthcare priority-setting process into a system is an intrinsically political act recognizing actors’ interests in what processes and criteria should be followed to allocate resources as well as recognizing actors’ involvement, roles, and responsibilities in the healthcare priority-setting process.^13-15^ However, the political process (policy formulation process) through which countries come to adopt explicit healthcare priority-setting processes largely remains unanalysed.^13,16^

 Kenya — a lower-middle income country in East Africa — is yet to achieve UHC with a UHC index of 51.5% signifying limited access to essential health services.^17^ Service coverage is also inequitable with a pro-rich distribution.^17^ In addition, at 2%,^17,18^ Kenya’s general government health expenditure as a percentage of the gross domestic product (GDP) is below the 5% of GDP required to achieve UHC in low- and middle-income countries (LMICs).^19^ On June 8, 2018, Kenya’s Ministry of Health (MoH) gazetted a new policy on the constitution of a Health Benefits Package Advisory Panel (HBPAP).^20^ HBPAP, a committee of 15 members (1 chairman and 14 members) and 2 joint secretaries, was introduced as a mechanism for conducting an explicit healthcare priority-setting process for health benefits package development in Kenya.^20^ A health benefits package outlines a specific set of health services for a defined population to be purchased from pooled resources.^6,21,22^

 The policy on HBPAP was a procedural policy as it sought to influence how and by whom the healthcare priority-setting process for health benefits package development was conducted at the national level in Kenya. A procedural policy refers to any course of action that changes how and by whom processes or functions of an organization or government are conducted.^23,24^ However, the political process that informed the HBPAP policy remains unclear. We therefore conducted this study to answer the following question: What was the political process that influenced the development and gazettement of the HBPAP policy idea in Kenya?

## Methods

###  Study Design

 We used a case-study approach to conduct a retrospective policy analysis of the political process that led to the gazettement of the policy idea on HBPAP. The case study method allows a detailed inquiry into the dynamics around a case which refers to a “*phenomenon within its real-life context.*”^25^ The case in this enquiry was the gazettement of the HBPAP policy idea. We used an interpretive epistemological approach to draw on participants’ perspectives and contextual factors^26,27^ to provide a rich explanation of why the HBPAP policy idea was gazetted.

###  Study Setting

 This study was conducted at the national level in Kenya where HBPAP was established. Kenya’s governance structure is devolved with administrative, fiscal and political functions divided amongst 1 national and 47 semi-independent county governments.^28^ Health is devolved between the national and county governments. At the national level, the MoH is the highest political office for the health sector. Its mandates include formulating health policies, building capacity, providing technical assistance and overseeing service delivery in tertiary public referral healthcare facilities.^28^

 Resources for Kenya’s health sector come from three main sources namely public (tax and public health insurance), private (household out-of-pocket payments and voluntary health insurance) and donors. In 2019, these sources contributed 46%, 35.5%, and 18.5% of the total health expenditure respectively.^29^ Purchasing, which refers to the transfer of pooled resources to healthcare providers for the provision of health services,^5^ is done through three models. The first model is the integrated public model where the MoH purchases services from tertiary public referral hospitals while the County Departments of Health purchase services from county public healthcare facilities namely community units, primary care facilities (dispensaries and health centres) and secondary referral facilities (primary care and secondary care hospitals). The second model is the public contract model where the National Health Insurance Fund (NHIF) — a state corporation — purchases services from both public and private (for-profit and not-for-profit) healthcare facilities. The third and final model is the private contract model where private health insurers purchase services from private healthcare facilities.^30,31^

###  Theoretical Framework

**Figure 1:**
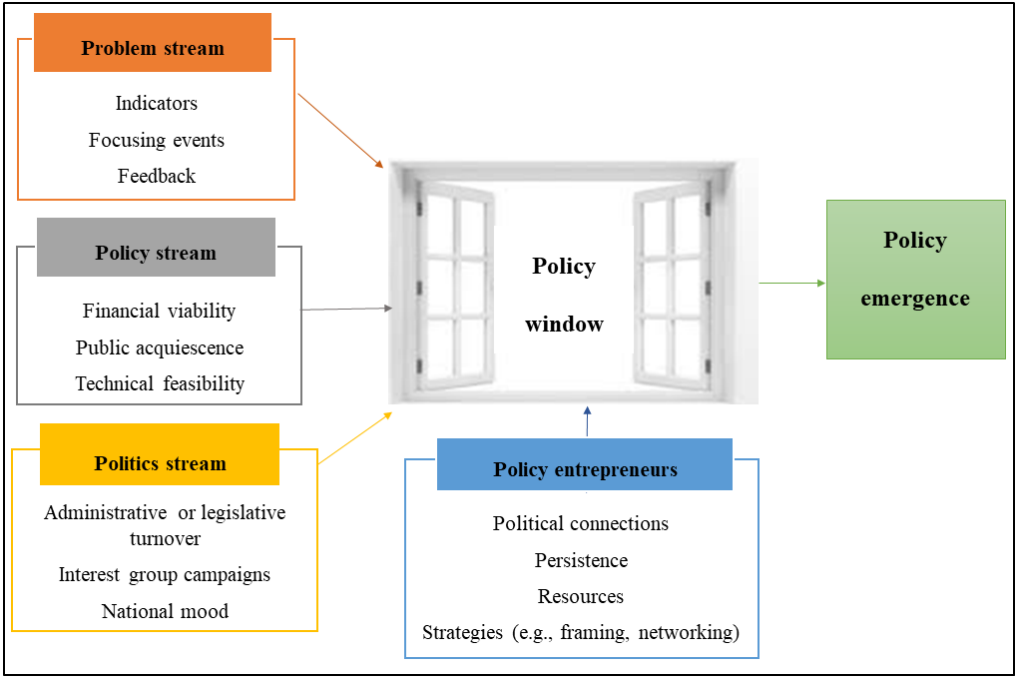
Kingdon’s Multiple Streams Theory.^34,35^

 We used Kingdon’s Multiple Streams Theory (Figure 1) given its demonstrable conceptual and empirical validity in explaining agenda-setting and other policy formulation stages through its broad application in multiple sectors, multiple levels of governance, and multiple countries.^32,33^ Agenda-setting refers to the process through which issues and potential policy solutions earn policy-makers’ attention leading to policy formulation.^34^

 The three streams in Kingdon’s theory are problem, policy, and politics. The problem stream refers to situations that deviate from what is considered normal or unrealized needs that require improvement through public (government) efforts.^34^ Problems become visible through indicators (measures of the level or severity of a problem), focusing events (unexpected occurrences such as crises or disasters that highlight a problem), and feedback (information given back on the performance of similar policies and programs).^34,35^

 The policy stream refers to potential solutions for addressing the problems.^34,35^ Policy solutions are developed by policy communities composed of individuals who are interested in influencing a specific policy area.^34^ Since policy communities often develop numerous policy solutions, several factors influence which solution might be considered for adoption by policy-makers. These factors include technical feasibility (whether the proposed solution works), public acquiescence (whether the mass public or attentive publics—individuals who are better informed and keenly interested in a particular issue than the general public—find the proposed solution acceptable), and financial viability (whether the proposed solution has acceptable cost implications given the existing budget).^34^

 The politics stream refers to the broader political context surrounding the policy under consideration. This stream is characterized by factors such as administrative or legislative turnover (changes in administration or legislation arising from campaigns, elections or nominations), national mood (the publics’ or elected government officials’ orientation towards issues), and interest group pressure (demands for action by groups such as civil societies).^34,35^

 The successful emergence of a policy follows coupling of the problem, policy, and politics streams. Coupling refers to the matching of a potential policy solution to an identified problem within favorable political conditions.^34,35^ Coupling occurs during a “policy window,” defined as a fleeting window of opportunity that expands or contracts the space for policy-making.^34,35^ A policy window opens due to compelling events in the problem or politics stream. Given the short nature of policy windows, timing is crucial: policy entrepreneurs must recognize them and act by introducing their preferred policy proposals when the political environment is receptive to change.^34,35^

 Policy entrepreneurs are actors within policy communities who are committed to engendering support for their preferred policy solutions from the public and policy-makers.^34,35^ They can be found inside or outside of government.^34,35^ The ability of policy entrepreneurs to achieve policy influence is determined by their access to key policy-makers. It is also determined by their persistence and willingness to invest resources (eg, time, money, and technical skills) into the process. Policy entrepreneurs can also influence policy by employing strategies such as: (*a*) framing (the structuring and presentation of information on problems or policy solutions to generate specific views, meanings, or perceptions^34,35^); (*b*) collecting evidence^36,37^; and (*c*) networking (engaging other relevant actors within policy communities to strengthen their likelihood of generating policy changes^36,37^).

 We used Kingdon’s theory to: (*a*) inform questions asked during data collection, (*b*) generate codes and themes during data analysis and, (*c*) synthesize findings on the political process that led to the gazettement of the HBPAP policy idea.

###  Data Collection

 We collected data through interviews and document reviews between July and September 2021.

####  In-depth Interviews

 We selected participants for in-depth interviews through purposive sampling. The purposive criterion was the participant’s known involvement in the political process for introducing the HBPAP policy. Participants were invited to the study via telephone and email – none declined participation. Prior to the interviews, participants reviewed the study’s information sheet and provided informed consent. The interviews were conducted via face-face at the participant’s place of work or via Zoom video conferencing.

 All interviews were conducted in English and recorded using an encrypted audio-recorder. The interviews lasted between 45 and 80 minutes. We used a semi-structured topic guide developed from the study’s theoretical framework to elicit participant’s views on the problems associated with healthcare priority-setting processes for health benefits packages as relevant to HBPAP’s main mandate. The interview guide also elicited participants’ views on alternative policy ideas that were considered alongside HBPAP; the political context surrounding the policy idea on HBPAP; and lastly, the policy windows and policy entrepreneurs that led to the gazettement of the HBPAP policy idea. We also took fieldnotes during the interviews to summarize discussion points when participants requested for the tape recorder to be switched off and as aids for critical reflection of emerging themes.

 We interviewed 20 participants (Table 1). We did not include additional participants since we had achieved theoretical saturation, that is, there was no new/additional information being obtained by the end of the 20th interview. This is in line a literature review that revealed that qualitative studies typically achieve saturation after between 9-17 in-depth interviews.^38^ We do not disclose participants’ demographic information to preserve their confidentiality and anonymity.

**Table 1:** List of Participants

**Category**	**Number**
Donor-supported technocrats	7
Local researchers	3
MoH technocrats	8
Semi-autonomous government agencies’ technocrats	2
**Total**	**20**

Abbreviation: MoH, Ministry of Health.

####  Document and Media Reviews

 We also collected data from documents to supplement and triangulate data obtained from interviews. We reviewed digital formats of documents that contained text and non-text data (such as images) from organizations such as the government, semi-autonomous government agencies, local research organizations, development partners, and mass media. These documents were identified by the study participants. They were also identified by the research team from online searches of websites that belong to organizations that are involved in macro-level healthcare priority-setting and from mass media. A full list of these documents is provided in Table S1 in Supplementary file 1.

###  Data Analysis

 Audio recorded data were transcribed verbatim into Microsoft Word. We reviewed each transcript for transcription accuracy against the respective audio-file and cleaned where necessary. We transferred fieldnotes to Microsoft word documents to prevent loss of data through forgetfulness. Each fieldnote was linked to the respective interview through dates and numbers.

 To facilitate data analysis, we uploaded the transcripts and digital formats of the retrieved documents (organizational reports and, texts and images from media) to NVivo software to facilitate thematic analysis using the Braun and Clarke approach.^39^ In phase 1, we immersed ourselves in the data contained in the transcripts and documents through reading and re-reading to engender familiarization. In phase 2, we coded data deductively drawing on the constructs outlined in the conceptual framework, that is, problems, policy solutions, politics, policy windows, and policy entrepreneurs (See Figure 1) which formed the coding framework. In phase 3, we generated a list of themes by identifying meaningful patterns within the coded data. In phase 4, we verified the quality of the themes by checking whether the themes reflected the patterns of meaning in the coded data. In phase 5, we applied the approved themes across the data sources to extract quotes, excerpts, and images that supported the themes. In phase 6, we produced a synthesis report on why the HBPAP policy idea was gazetted. This report was revised and approved by all the authors.

###  Trustworthiness

 We built trustworthiness in the study findings by applying several data collection methods [triangulation of methods] and by interviewing different participants to discover different perspectives [triangulation of data sources]. As a team, we also held peer debriefing sessions to critique the interview topic guide as well as emerging codes and themes to ensure that these were based on the data.

###  Reflexivity

 All authors have supported national level policy processes across different LMICs. This participation informed authors’ selection of the study topic, study design, participants, and data collection methods. In Kenya specifically, one of the authors has been involved in policy formulation processes at the national level which strengthened data collection by facilitating knowledge, identification, and access to the participants known to be involved in national policy processes and by facilitating access to documents relevant to the study.

## Results

 In this section, we discuss the events in the political process that led to the gazettement of the HBPAP policy idea based on interviews with study participants and, document (organizational and media) reviews.

###  Policy Actors

 The political process that led to the gazettement of the HBPAP policy idea did not occur in public spaces with societal actors. Instead, it occurred in closed-door meetings involving technical and political actors, though the attentive publics consisting of national and county stakeholders in the health financing intergovernmental coordinating committee also had some influence (Table 2). Technical actors (technocrats) played key roles in defining the problem and policy streams. Political actors shaped the political stream by prioritizing UHC, whilst the attentive publics were consulted during the development of potential policy solutions as discussed below.

**Table 2:** Actors and Their Roles in the Emergence of the Policy Idea on HBPAP

**Actor**	**Type of Actor**	**Role**
President Uhuru Kenyatta	Political	Served as Kenya’s president from 2013. Declared UHC as one of his big 4 presidential agenda for his 2nd term (2017-2022). Appointed Dr. Cleopa Mailu and Mrs. Sicily Kariuki as the Cabinet Secretaries for Health in 2015 and 2018, respectively.
Dr. Cleopa Mailu	Political	Served as the Cabinet Secretary for Health between 2015 and 2017. Formed the 2015 TWG to develop Kenya’s health financing strategy.
Mrs. Sicily Kariuki	Political	Served as the Cabinet Secretary for Health from January 2018. Mandated to implement the Presidential Agenda on UHC. Gazetted HBPAP in June 2018, and appointed its members.
UHC coordination department in the MoH	Technical	Formed by Mrs. Sicily Kariuki to formulate and implement UHC reforms. Developed the terms of reference for HBPAP.
MoH technocrats	Technical	Developed and implemented health financing and UHC reforms. Were part of the 2015 TWG that identified health financing problems in Kenya and proposed potential policy solutions.
Local researchers	Technical	Provided technical support and research evidence for health financing and UHC reforms. Were part of the 2015 TWG that identified health financing problems in Kenya and proposed potential policy solutions.
Donor-supported Kenyan technical advisors	Technical	Provided technical support for health financing and UHC reforms. Were part of the 2015 TWG that identified health financing problems in Kenya and proposed potential policy solutions.
NHIF technocrats	Technical	Were part of the 2015 TWG that identified health financing problems in Kenya and proposed potential policy solutions.
Private health sector (Christian Health Association of Kenya, Kenya Health Federation) technocrats	Technical	Were part of the 2015 TWG that identified health financing problems in Kenya and proposed potential policy solutions.
Intergovernmental coordinating committee	Technical	Consisted of national and county-level stakeholders who were consulted during the development of the health financing strategy.

Abbreviations: MoH, Ministry of Health; UHC, universal health coverage; TWG, Technical Working Group; HBPAP, Health Benefits Package Advisory Panel; NHIF, National Health Insurance Fund. Source: interviews and document reviews.

 In the period of focus, the political actors included President Uhuru Kenyatta who was the holder of the highest political office in Kenya and, Dr. Cleopa Mailu and Mrs. Sicily Kariuki who were the Cabinet Secretaries for Health (analogous to Ministers for Health) or the holders of the highest political office in the MoH. Technocrats include health economists, health financing experts, health policy analysts, and health systems experts from various organizational bases. The MoH technocrats are civil servants with technical training and expertise in developing policies and supporting MoH’s functions. They have a long history of working with donor-supported Kenyan technical advisors. The donors include multilateral agencies such as World Bank and bilateral agencies such as United States Agency for International Development, Japan International Cooperation Agency, and German Agency for International Cooperation. MoH technocrats also have a long history of working with local researchers producing relevant health financing research. The NHIF, a semi-autonomous government agency, is the largest public health insurer, purchasing services from public and private healthcare facilities. The private health sector comprises both for-profit and not-for-profit healthcare providers.

###  The Problem Stream: Fragmented and Implicit Healthcare Priority-Setting Processes and, Unaffordable, Unsustainable, and Wasteful Benefits Packages

**Figure 2:**
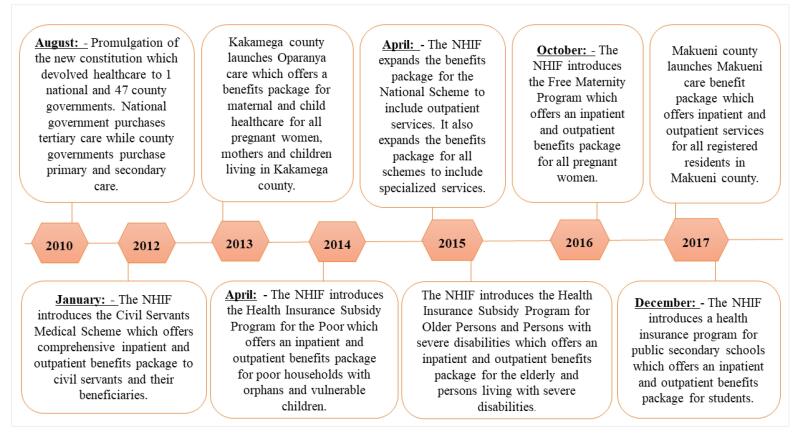
A Timeline Showing Introduction of Multiple Health Benefits Packages by Different Purchasers in Kenya’s Public Health Sector. Abbreviation: NHIF, National Health Insurance Fund.

 Technical actors directly involved in health financing and UHC reforms since 2015 found that healthcare priority-setting processes for health benefits package development in Kenya were fragmented and implicit (non-transparent, non-inclusive and non-evidence based) based on the evidence obtained from situation analyses of Kenya’s health financing architecture.^40-43^ The healthcare priority-setting processes were fragmented because they were conducted by different priority-setting bodies (Table S2 in Supplementary file 1). Since 2010, this fragmentation had led to the introduction of multiple health benefits packages with different service entitlements for different population groups (Figure 2).

 The existing healthcare priority-setting processes were also non-transparent and non-inclusive due to poor stakeholder involvement.

 “*Decisions on which services Kenyans can access are very fragmented and made in silos such that only those involved in those processes are aware what processes are used. We just hear that some new drugs or vaccines have been introduced, but how did they get there? What processes did NHIF use to introduce its benefits packages? It is like a black box in decision-making” *(Participant 3, MoH technocrat).

 Lastly, the healthcare priority-setting processes were non-evidence based given the inadequate use of explicit priority-setting criteria such as cost-effectiveness, feasibility, and affordability. Instead, these processes were driven by historical patterns of resource allocation and stakeholder interests. The lack of evidence-based healthcare priority-setting processes had led to broad and poorly defined benefits packages that were wasteful, unaffordable, and unsustainable. This was concerning given the chronic underfunding of Kenya’s health sector.^41-43^

 “*The situation analysis showed us that the process of developing the multiple benefit packages was not guided by any systematic or evidence-based process. The process was neither transparent nor inclusive. The symptoms of that broken system were benefit packages that were not affordable or sustainable” *(Participant 1, local researcher).

 While there were indicators and feedback from situation analyses highlighting the problem stream, there were no focusing events that suddenly highlighted these issues in Kenya.

###  The Policy Stream: Establishment of an Independent Expert Panel as a Potential Policy Solution 

 The technical actors directly involved in health financing and UHC reforms since 2015 recognized that addressing the problems facing existing healthcare priority-setting processes would enable Kenya to progress towards UHC. This recognition was based on other countries’ experience of attaining UHC such as Thailand.^40-43^ These technocrats recommended several policy ideas as potential solutions to the problems. One such policy idea was the establishment of a single funding pool to consolidate revenue across the different purchasers thereby reducing fragmentation. However, this proposal was wrought with feasibility concerns due to stakeholder interests and lack of a framework for consolidating the pools.^41-43^

 Another policy idea recommended by these technocrats was the establishment of an independent expert panel to harmonize the healthcare priority-setting process for health benefits package development across all purchasers thus reducing fragmentation. This panel would define a basic health benefits package for all purchasers in Kenya by using an explicit and evidence-based approach.^40-43^

 “*When we were writing the health financing strategy in 2015, there was a suggestion that we needed an independent panel of experts to develop a harmonized benefit package using an independent, explicit, evidenced, and inclusive process*” (Participant 2,Donor-supported technocrat).

 While majority of the technocrats supported the policy idea of establishing an independent expert panel, a few others such as the NHIF and some MoH technocrats opposed this policy idea because they considered development of health benefits packages as one of their organization’s mandates.

 “*The contestation was whether the package should be developed by an independent expert panel, the MoH or NHIF. There were those MoH officials who insisted that the MoH should be the one to propose a benefit package. Then, from a historical perspective, NHIF has been defining services. Divorcing benefits from its roles was very thorny”* (Participant 5, Donor-supported technocrat).

 Given this contestation, the technocrats compared the policy idea of establishing an independent expert panel to the policy idea of having the MoH and other purchasers become the main priority-setting bodies for designing the basic health benefits package (Table S3 in Supplementary file 1). These comparisons were made in consultation with attentive publics namely national and county stakeholders through the health financing intergovernmental coordinating committee. From this comparison, there was acquiescence from the attentive publics for the policy idea of establishing an independent expert panel for health benefits package development. The country also had the technical expertise to build a dedicated team for the panel. However, the administrative and operational costs of managing a panel were thought to be prohibitive thus undermining its financial viability.^42,43^

 “*The panel was a great shift towards systematic priority-setting for our country. It was uniquely designed to ensure that the process of setting priorities was open and transparent which was different from what was being done in the past” *(Participant 3, MoH technocrat).

###  The Politics Stream: Changing Political Landscape

 The political landscape of the period between 2010 when fragmentation of healthcare priority-setting processes for benefits packages began (Figure 2) to 2018 when the HBPAP policy idea was gazetted, was characterized by changes in national mood and, administrative and legislative turnover.

###  National Mood

 In 2010, Kenya promulgated a new constitution that was approved by 67% of Kenyan voters.^44^ This new constitution recognized health as a basic human right. It also recognized that every Kenyan had a right to the highest standard of affordable healthcare.^28^ The recognition of these health-related rights in Kenya’s supreme law represented national interest in health. This national interest further generated technical actors’ interest in defining a basic package of health services to enable access to healthcare as outlined in the constitution.

 “*The 2010 constitution led us to this very clear realization that there needs to be a lot of effort in trying to determine and ensure provision of a set of services that are crucial to the people. This discussion led to the whole concept of the basic benefits package”* (Participant 8, MoH technocrat).

 The national mood was ultimately shaped by the political prioritization of UHC. In 2017, President Uhuru Kenyatta declared UHC as one of the four pillars for socio-economic development.^45^ The President’s declaration on UHC was a symbolic act of the leading political party’s standpoint on matters related to health which created a positive national mood for UHC that influenced health system stakeholders.

 “*The President declared that he would like Kenya to attain UHC during this tenure. This very high-level policy statement cascaded to the Cabinet Secretary and the technocrats at the ministry who had to look for different ways of translating that directive into action” *(Participant 5, MoH technocrat).

###  Administrative and Legislative Turnover

 In 2010, the biggest legislative turnover took place in Kenya when the new constitution was promulgated. This led to the devolution of revenue collection, pooling, and purchasing of health services between 1 national and 47 county governments which contributed to the fragmentation of healthcare priority-setting processes as these organizations had different processes for developing benefits packages.

 “*It was really with devolution that fragmentation in the health sector became more evident. There were 47 county government pools and 1 national pool each responsible for health service delivery at different levels”* (Participant 2, MoH technocrat).

 Several administrative changes also shaped the political context for the policy and problem streams. In 2013, President Uhuru Kenyatta was successfully elected as the first president under the new constitution. Given his interest in affordable healthcare, he introduced several reforms such as user-fee removal in primary healthcare facilities and free maternity care in all public hospitals. While these reforms were meant to increase access to care, they inadvertently contributed to fragmentation of benefits packages in the country. It was also unclear which priority-setting processes had informed these new benefits.

 “*Following the first term of Jubilee, we had waiver of user fees, Linda Mama for pregnant women to access ante-natal and maternity services and the health insurance subsidy program. However, it was paradoxical that we did not know how these decisions were arrived at”* (Participant 1, Technocrat from Semi-autonomous government agency).

 In 2015, Dr. Cleopa Mailu was appointed as the new Cabinet Secretary for Health by President Uhuru Kenyatta. Dr. Mailu established the 2015 multi-stakeholder Technical Working Group (TWG) to develop a health financing strategy for Kenya. This TWG consisted of technocrats from different organizations (Table 2) who not only identified the issues outlined in the problems stream but also recommended potential policy solutions for the identified problems.

 In 2017, Mr. Uhuru Kenyatta was re-elected as the president for a second term. He declared UHC as one of his main agenda which influenced the national mood towards UHC reforms. In January 2018, President Uhuru Kenyatta reshuffled his cabinet and appointed a new Cabinet Secretary for Health, Mrs. Sicily Kariuki, who then established a UHC coordination department to formulate and implement UHC.

 “*In early 2018, the new cabinet secretary identified a small group of people within the ministry who were going to help her deliver UHC. Then she went ahead and created a department for them” *(Participant 3, MoH technocrat).

###  Emergence of a Policy Window, Coupling of the Streams, and the Role of Policy Entrepreneurs

 Despite being initially proposed in 2015, the policy idea of establishing an independent expert panel was not adopted because of unfavourable political conditions.

 “*In 2015, we came up with the idea of an independent panel that would be designing and reviewing the benefits package every two years. But at that point, it was not taken forward because there was no political momentum”* (Participant 6, Donor-supported technocrat).

 However, in 2017, there was a shift in the political momentum when President Uhuru Kenyatta politically prioritized UHC as one of his four main agenda. The prioritization of UHC created a policy window that a group of technocrats, turned policy entrepreneurs, seized to advocate for policies that would help the President achieve his ambition. This group of technocrats had been directly involved in defining the problem and policy streams since 2015. One of their policy recommendations was the establishment of an independent expert panel to develop a basic health benefits package.

 “*In December 2017, the President indicated that UHC was one of his Big Four Agenda in his second term. That was a water shed moment because it opened a window that had not existed before for us to push for proposals for health financing reforms that had been on-going for the last decade” *(Participant 5, MoH technocrat).

 Recognizing their shared individual and organizational ideologies and interests in health financing and UHC reforms, the technocrats turned policy entrepreneurs deliberately engaged and interacted with each other in a network since 2015. In these networks, the policy entrepreneurs utilized their technical expertise and professional experience to develop policy ideas for UHC and health financing reforms, including the policy idea of an independent expert panel for health benefits package development. They outlined these policy ideas in presentations, health financing strategies, and cabinet memoranda.

 “*We had informal discussions with some MoH officials and development partners where we were discussing what could be done in terms of health reforms. We developed PowerPoints and draft cabinet memo to lobby for change”* (Participant 3, local researcher).

 In April 2018, some MoH officials, who were part of the network of policy entrepreneurs, took advantage of an impromptu meeting with the Cabinet Secretary, Mrs. Sicily Kariuki, to highlight potential policy ideas for achieving UHC. Among the policy ideas presented to the Cabinet Secretary, was the idea of establishing an independent expert panel for health benefits package development.

 “*An opportunity appeared when the Cabinet Secretary came in requesting for a meeting on UHC. The MoH officials presented the cabinet memo to her, and this is what convinced her to establish the panel to change how services were being purchased within the country in the journey towards UHC”* (Participant 6, MoH technocrat).

 The network of policy entrepreneurs also employed framing of the problem and policy streams (Table 3) as a strategy to elicit support from the policy-makers. Some of these framings rode on the President’s promise for UHC and the Cabinet Secretary’s mandate to implement the President’s Agenda on UHC. To strengthen these frames, the policy entrepreneurs drew on the evidence obtained from the situation analyses. They also drew on international evidence to demonstrate that countries such as Thailand had successfully introduced independent experts panels for healthcare priority-setting for health benefits package development.^40^

**Table 3:** Frames Used by Policy Entrepreneurs to Elicit Policy-Makers’ Support

**Frame**	**Examples of illustrative quotes**
**Framing of the Fragmentation of Priority-Setting Processes and Benefits Packages**	
Source of inefficiency	*“Fragmentation of priority-setting processes and of benefit packages increased inefficiencies in the health system through resource leakages”* (Document excerpt).
A bottle neck in UHC implementation	*“One of the biggest challenges in the implementation of UHC was the lack of a standard health benefits package”* (Participant 8, MoH technocrat).
**Framing of the Independent Experts Panel**	
A way of harmonizing fragmented priority-setting processes	*“Since the priority-setting mechanisms were very fragmented and siloed, we needed a mechanism for bringing all these together to have a uniform way of doing things. This would be achieved through an independent panel”* (Participant 1, MoH technocrat).
A strategy for operationalizing and fulfilling the presidential agenda on UHC	*“To operationalize UHC within Kenya, we showed that there was a need to define a health benefits package. The panel would define this benefits package therefore offer an instrument to realize the UHC objective”* (Participant 4, Donor-supported technocrat).
A strategy for developing a health benefit package transparently and accountably	*“A health benefits authority should be established with a structured process for priority-setting to explicitly define a package of services in a transparent and accountable manner”* (Document excerpt).
A mechanism for strategic use of funds and maximization of health outcomes	*“The panel was a process to ensure that priorities are set, and the available resources are used strategically to get the maximum outcome for a larger population”* (Participant 3, local researcher).
A mechanism for defining a fiscally sustainable benefits package	*“The panel was going to methodologically define one explicit benefits package that the government was going to commit to provide by considering what services the government could afford” *(Participant 7, Donor-supported technocrat).
A process that enhances the responsiveness of the benefits package to population health needs	*“There was a need to define explicitly what essential benefits Kenyans would access under UHC while being cognizant of health as a constitutional right, our socio-economic reality and major causes of disease burden in the country. The best way to do this was to set up an independent expert panel”* (Participant 4, MoH technocrat).
An inclusive platform that is owned and led by the people	*“It was very important to have an inclusive team that represents different sectors for stakeholder buy-in. We wanted it to be a process that was driven and owned by the people and not the ministry. It was a way to get more people on the table to contribute” *(Participant 7, MoH technocrat).
A process that guarantees independence and is insulated from influence by internal (bureaucratic) and external actor interests	*“The panel was going be an independent body of highly technical people responsible for developing the health benefits package. Its independence would ensure there was no influence from the Ministry or NHIF on how the process is done”* (Participant 2, MoH technocrat).

Abbreviations: MoH, Ministry of Health; UHC, universal health coverage; NHIF, National Health Insurance Fund.

**Figure 3:**
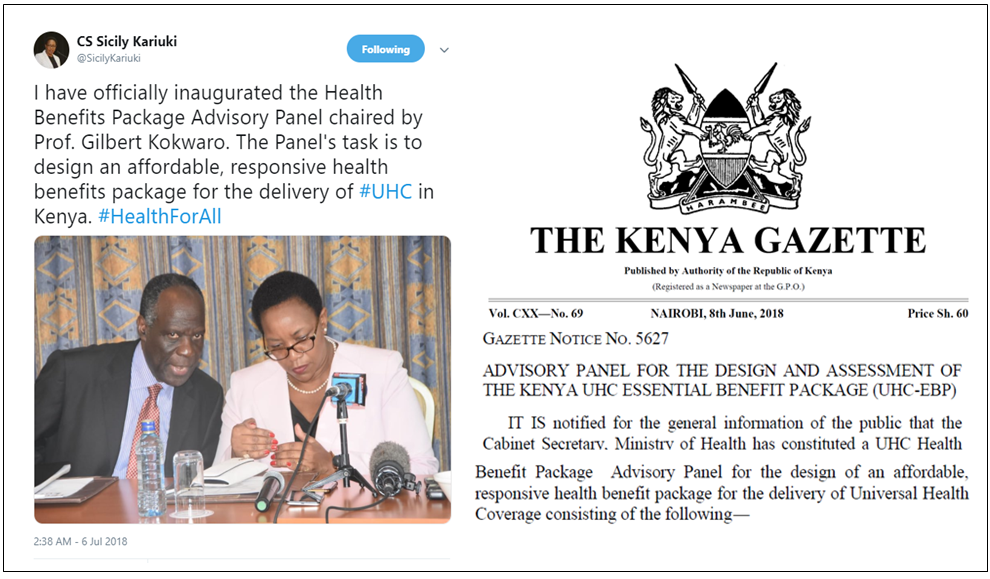
Gazettement of the Health Benefits Package Advisory Panel Policy. Abbreviations: MoH, Ministry of Health; UHC, universal health coverage.

 The policy entrepreneurs also utilized the new political access to Mrs. Sicily Kariuki, which was created during the impromptu meeting, to continue lobbying for the creation of an independent expert panel. Their persistence and persuasive framing paid off when, on June 8, 2018, Mrs. Sicily Kariuki gazetted the HBPAP policy idea and appointed its members (Figure 3).^20^ Mrs. Sicily Kariuki worked closely with the technocrats in the UHC coordination department and the policy entrepreneurs to develop the substantive content of the policy namely HBPAP’s roles, composition, and mandates.

 “*Through both formal and informal meetings, we were able to inform the Cabinet Secretary on what it would take to truly attain UHC. It is those discussions that led her to gazette the panel which consisted of experts drawn from the entire sector*” (Participant 5, MoH technocrat).

## Discussion

 In this study, we used Kingdon’s multiple streams theory to examine the political process that led to the gazettement of the HBPAP policy idea in Kenya. The HBPAP policy is an example of a procedural policy^23,24^ as it sought to change how and by whom the healthcare priority-setting process for health benefits package development would be conducted in Kenya. Consistent with Kingdon’s theory, the HBPAP policy idea was gazetted following the timely action of a network of policy entrepreneurs who matched the HBPAP policy idea to the identified problems of fragmented and implicit priority-setting processes and, unaffordable, unsustainable, and wasteful benefits packages when a policy window opened in the political stream. We discuss the significance of these findings against international literature.

 In this study, a policy window opened spontaneously in the political stream following the re-election of the President and the appointment of a new minister for health who were committed to UHC. Administrative changes open policy windows by creating the political impetus required for consideration of policy proposals. For example, in California, the election of Arnold Schwarzenegger, an ardent supporter of health and fitness activities, as a governor opened a critical policy window for tobacco control policies.^46^ In the Wallonia- Brussels Federation in Belgium, the election of a new government in 2004 and the appointment of a doctor to oversee preventive medicine, an uncommon occurrence, created a policy window for the adoption of a hearing screening program for newborns.^47^ Lastly, in Canada, the appointment of a new cabinet minister created a policy window that led to the approval of the national health insurance policy.^48^

 Our study demonstrates the crucial role of policy entrepreneurs in coupling the streams during a policy window. To achieve this, these policy entrepreneurs employed several strategies. Firstly, they were adequately prepared with evidence of the issues in the problem stream and evidence of the viability of potential policy solutions based on situation analyses and other countries’ experiences, respectively. The availability of these evidence strengthened policy-makers’ recognition of the problem and acceptance of the policy solutions. Similar findings have been reported in a Canadian healthcare centre where policy entrepreneurs generated stakeholders’ acquiescence by providing empirical evidence of the successful introduction of Program Budgeting and Marginal analysis as an approach for explicit healthcare priority-setting in other contexts.^16^ Policy entrepreneurs who are adequately prepared with evidence on the problem and policy streams can effectively influence policy change.^37,49^

 Secondly, recognizing common interests, the group of Kenyan technocrats who came to act as policy entrepreneurs deliberately engaged and interacted with each other in a network. For policy entrepreneurs, networks offer multiple benefits. For example, networks increase available resources.^37,50^ In our study, the network of policy entrepreneurs increased the technical and professional expertise required to support the development of the HBPAP policy. Similarly, in Lebanon, academic researchers built a network of policy entrepreneurs with civil society organisations, non-governmental organisations, and the media which increased their resources for advocacy campaigns for the tobacco control policy.^51^ Networks also build alliances of policy supporters thereby increasing the prominence of policy entrepreneurs in the policy-making environment.^36,50^ In our study, the network led to a greater coalition of supporters for the problem and policy streams from the MoH, Development partners, and Research organizations. Similarly, the United Nations Women, an international policy entrepreneur, worked collectively with other actors from government and non-governmental agencies to generate greater political support for the problem of violence against women.^52^

 Thirdly, policy entrepreneurs in our study used framing to draw more support from policy-makers. Framing raises the political profile of a problem or policy solution by evoking policy-makers’ views and judgements which enables the problem or policy to ascend to the policy-makers’ agenda.^34,37,53^ Framing can be achieved by strategically linking ideologically congruent frames about a problem or policy solution with wider political and socio-economic ideologies or commitments at individual, sub-national, national and/ or international level.^50,53^ In Kenya, policy entrepreneurs used framing to strategically link the problem and policy streams to the national commitment for UHC thereby increasing the political priority of these streams. In countries such as Nepal, by framing gender based violence as a human rights issue, policy entrepreneurs were able to link it to the country’s new constitution and to the interests of the Prime Minister’s office leading to the approval and adoption of the bill on gender based violence.^54^ Globally, policy entrepreneurs were able to raise the global priority for cervical cancer and violence against women by linking them to non-communicable diseases^55^ and COVID-19 pandemic lockdown restrictions,^52^ respectively.

 Lastly, policy entrepreneurs in our study utilized their proximal access to the Cabinet secretary to lobby for their preferred policy solution which enabled them to influence the policy process. Similarly, in Lebanon, policy entrepreneurs leveraged their political connections to lobby for tobacco control policies.^51^ Literature shows that the proximity of a policy entrepreneur to policy-makers impacts on their activities and/ or effectiveness in bringing about policy changes.^50,56^

 This study offers important insights on the roles that different health systems stakeholders in Kenya and other LMICs can play in the political process for developing and gazetting policies related to healthcare priority-setting processes. For the technocrats, they can: (*a*) identify problems related to healthcare priority-setting processes by conducting situational analyses of their country’s health financing architecture; (*b*) develop potential policy solutions to the identified problems; and (*c*) act as policy entrepreneurs during a policy window by networking with other technocrats, framing, marshalling evidence, and leveraging political connections to raise the political profile of policy problems and potential solutions. For policy-makers, administrative changes and political statements can create windows of opportunity for the emergence of new policies on healthcare priority-setting. Lastly, for the lay and attentive publics, their inputs can influence legislative changes and the development of policy solutions respectively which can shape the process of development and gazettement of policies on healthcare priority-setting.

###  Limitations

 Recall bias is a potential limitation in this study given the retrospective nature of the study. However, by including document and media reviews, which are important historical accounts of past events,^57^ we minimized this bias. Another potential limitation is social desirability bias whereby participants alter responses in the belief that these would make the responses more acceptable. However, by triangulating data sources and methods, we strengthened the trustworthiness of the findings. While we purposively selected stakeholders involved in macro-level policy development, we did not interview county level stakeholders which poses a potential limitation of failing to capture their perceptions. Future studies should therefore consider including subnational stakeholders as this may generate greater insights. Lastly, the involvement of one of the authors in previous policy formulation processes in Kenya may have biased the interviews and analysis, but this was mitigated through document and media reviews, and peer debriefing sessions among the authors.

## Conclusion

 Applying Kingdon’s theory in this study was valuable in explaining the political process that led to the gazettement of the procedural policy on HBPAP. Technocrats from different organizational bases (MoH, Local research organizations, Development partners, NHIF, and the Private Sector) played key roles in defining the problem and policy streams. Political actors such as the President and the Cabinet Secretary for Health shaped the national mood in the political stream by prioritizing UHC. Attentive publics such the health financing intergovernmental coordinating committee were consulted during the development of potential policy solutions. A group of technocrats, turned policy entrepreneurs, played a crucial role in coupling the problem, policy, and political streams during a policy window that was created by the political prioritization of UHC. To achieve coupling, these policy entrepreneurs employed strategies such as working in networks; persuasive framing of problems and policy proposals; utilizing political connections, and marshalling of evidence on problems and policy streams. These insights can be useful to other countries seeking to introduce procedural policies on healthcare priority-setting processes for health benefits package development. This study also offers useful insights to local and international academic communities on the suitability of policy analysis theories in examining political processes for formulating procedural policies for healthcare priority-setting processes which remain limited.

## Ethical issues

 Ethical approval for this study was obtained from the London School of Hygiene and Tropical Medicine Ethics Committee (Reference Number: 25640) and the Kenya Medical Research Institute Scientific and Ethics Review Unit, Nairobi, Kenya (Reference Number: KEMRI/SERU/CGMR-C/185/4018).

## Competing interests

 Authors declare that they have no competing interests.

## Disclaimer

 The views expressed in the paper are for the authors and not for the organizations they represent.

## Supplementary files

Supplementary file 1 contains Tables S1-S3.

## References

[1] United Nations Development Program. Transforming our World: The 2030 Agenda for Sustainable Development. New York: United Nations; 2017. https://sustainabledevelopment.un.org/content/documents/21252030%20Agenda%20for%20Sustainable%20Development%20web.pdf.

[2] World Health Organization (WHO). WHA58.33 Sustainable Health Financing, Universal Coverage and Social Health Insurance. Geneva: WHO; 2005.

[3] World Health Organization (WHO). Making Fair Choices on the Path to Universal Health Coverage: Final Report of the WHO Consultative Group on Equity and Universal Health Coverage. Geneva: WHO; 2014.

[4] World Health Organization (WHO). Arguing for Universal Health Coverage. Geneva: WHO; 2013.

[5] World Health Organization (WHO). Health Systems Financing: The Path to Universal Coverage. Geneva: WHO; 2010. 10.2471/BLT.10.078741 PMC2878164 20539847

[6] Bobadilla JL, Cowley P, Musgrove P, Saxenian H (1994). Design, content and financing of an essential national package of health services. Bull World Health Organ.

[7] Derakhshani N, Doshmangir L, Ahmadi A, Fakhri A, Sadeghi-Bazargani H, Gordeev VS (2020). Monitoring process barriers and enablers towards universal health coverage within the sustainable development goals: a systematic review and content analysis. Clinicoecon Outcomes Res.

[8] Glassman A, Chalkidou K. Priority-Setting in Health: Building Institutions for Smarter Public Spending. Washington, DC: Center for Global Development; 2012.

[9] Coulter A, Ham C. The Global Challenge of Health Care Rationing. Buckingham, Philadelphia: Open University Press; 2000.

[10] McKneally MF, Dickens BM, Meslin EM, Singer PA (1997). Bioethics for clinicians: 13 Resource allocation. CMAJ.

[11] Chalkidou K, Glassman A, Marten R (2016). Priority-setting for achieving universal health coverage. Bull World Health Organ.

[12] World Health Organization (WHO). Health Intervention and Technology Assessment in Support of Universal Health Coverage (WHA67.23). WHO; 2014. http://apps.who.int/gb/ebwha/pdf_files/wha67/a67_r23-en.pdf.

[13] Smith N, Mitton C, Davidson A, Williams I (2014). A politics of priority setting: Ideas, interests and institutions in healthcare resource allocation. Public Policy Adm.

[14] Hauck K, Smith PC, Goddard M. The Economics of Priority Setting for Health Care: A Literature Review. Washington, DC: World Bank; 2004.

[15] Ham C, Glenn R. Reasonable Rationing: International Experience of Priority Setting in Health Care. Maidenhead, Philadelphia: Open University Press; 2003.

[16] Smith N, Mitton C, Dowling L, Hiltz MA, Campbell M, Gujar SA (2015). Introducing new priority setting and resource allocation processes in a Canadian healthcare organization: a case study analysis informed by multiple streams theory. Int J Health Policy Manag.

[17] Barasa E, Nguhiu P, McIntyre D (2018). Measuring progress towards sustainable development goal 38 on universal health coverage in Kenya. BMJ Glob Health.

[18] World Health Organization (WHO). Global Health Expenditure Database: NHA Indicators. WHO; 2022. https://apps.who.int/nha/database/ViewData/Indicators/en. Accessed December 2022.

[19] McIntyre D, Meheus F, Røttingen JA (2017). What level of domestic government health expenditure should we aspire to for universal health coverage?. Health Econ Policy Law.

[20] The Kenya Gazette. Gazette Notice No. 5627: Advisory Panel for the Design and Assessment of the Kenya UHC Essential Benefit Package (UHC-EBP). National Council for Law Reporting (Kenya Law), Republic of Kenya; 2018. http://kenyalaw.org/kenya_gazette/gazette/volume/MTgwMw--/Vol.CXX-No.69.

[21] Glassman A, Giedion U, Smith PC. What’s in, What’s out? Designing Benefits for Universal Health Coverage. Washington, DC: Center for Global Development; 2017.

[22] Glassman A, Giedion U, Sakuma Y, Smith PC (2016). Defining a health benefits package: what are the necessary processes?. Health Syst Reform.

[23] Howlett M. Policy tools and their role in policy formulation: Dealing with procedural and substantive instruments. In: Handbook of Policy Formulation. Edward Elgar Publishing; 2017.

[24] The Commonwealth of Learning (COL). E7: Policy Analysis and Implementation. Module 6 - Instruments of Government Policy. Vancouver, Canada: COL; 2012.

[25] Yin RK. Case Study Research: Design and Methods. New Delhi: SAGE Publications; 2003.

[26] Klein HK, Myers MD (1999). A set of principles for conducting and evaluating interpretive field studies in information systems. MIS Q.

[27] Walsham G (1995). Interpretive case studies in IS research: nature and method. Eur J Inf Syst.

[28] The Republic of Kenya. The Constitution of Kenya. National Council for Law Reporting (Kenya Law); 2010:1-194. http://kenyalaw.org/lex/actview.xql?actid=Const2010.

[29] World Health Organization (WHO). Global Health Expenditure Database - Health Expenditure Profile for Kenya. WHO; 2019. https://apps.who.int/nha/database/country_profile/Index/en. Accessed March 18, 2022.

[30] Mbau R, Barasa E, Munge K, Mulupi S, Nguhiu PK, Chuma J (2018). A critical analysis of health care purchasing arrangements in Kenya: a case study of the county departments of health. Int J Health Plann Manage.

[31] Munge K, Mulupi S, Chuma J. A Critical Analysis of the Purchasing Arrangements in Kenya: The Case of the National Hospital Insurance Fund, Private and Community-Based Health Insurance. Working Paper 7. London: RESYST; 2015. 10.15171/ijhpm.2017.81 PMC5890069 29524953

[32] Jones MD, Peterson HL, Pierce JJ (2016). A river runs through it: a multiple streams meta-review. Policy Stud J.

[33] Zahariadis N. The multiple streams framework: structure, limitations, prospects. In: Sabatier PA, ed. Theories of the Policy Process. United States of America: Westview Press; 2007:65-92.

[34] Kingdon JW. How do issues get on public policy agendas. In: Sociology and the Public Agenda. Newbury Park: SAGE Publications; 1993:40-53.

[35] Kingdon JW. Agendas, Alternatives, and Public Policies. Vol 45. Boston: Little, Brown; 1984.

[36] Mintrom M, Vergari S (1996). Advocacy coalitions, policy entrepreneurs, and policy change. Policy Stud J.

[37] Mintrom M (2019). So you want to be a policy entrepreneur?. Policy Des Pract.

[38] Hennink M, Kaiser BN (2022). Sample sizes for saturation in qualitative research: a systematic review of empirical tests. Soc Sci Med.

[39] Braun V, Clarke V (2006). Using thematic analysis in psychology. Qual Res Psychol.

[40] Republic of Kenya. Cabinet Memorandum: Roadmap to Attain Universal Health Coverage in Kenya by 2022. Nairobi: Republic of Kenya; 2018.

[41] Ministry of Health. Draft Kenya Health Financing Strategy 2016-2030. Nairobi: Ministry of Health; 2017:129.

[42] Ministry of Health. 3rd Draft Health Financing Strategy 2016-2030. Nairobi: Ministry of Health; 2016:63.

[43] Technical Working Group. 1st Draft Kenya Health Financing Strategy 2015-2030. Nairobi: Ministry of Health; 2015:55.

[44] Macharia J, Obulutsa G. Kenya Votes ‘Yes’ to New Constitution. 5th ed. Reuters; 2010.

[45] The Executive Office of the President. The Big 4 Agenda: Fast Tracking our Vision Through a 5-Year Development Plan Under 4 Key Pillars. Government of Kenya; 2017. https://big4.delivery.go.ke/.

[46] Blackman VS (2005). Putting policy theory to work: tobacco control in California. Policy Polit NursPract.

[47] Vos B, Lagasse R, Levêque A (2014). Putting newborn hearing screening on the political agenda in Belgium: local initiatives toward a community programme - a qualitative study. Health Res Policy Syst.

[48] Blankenau J (2001). The fate of national health insurance in Canada and the United States: a multiple streams explanation. Policy Stud Jl.

[49] Abiola SE, Colgrove J, Mello MM (2013). The politics of HPV vaccination policy formation in the United States. J Health Polit Policy Law.

[50] Gunn A. Policy entrepreneurs and policy formulation. In: Handbook of Policy Formulation. Edward Elgar Publishing; 2017.

[51] Nakkash RT, Torossian L, El Hajj T, Khalil J, Afifi RA (2018). The passage of tobacco control law 174 in Lebanon: reflections on the problem, policies and politics. Health Policy Plan.

[52] Mintrom M, True J (2022). COVID-19 as a policy window: policy entrepreneurs responding to violence against women. Policy Soc.

[53] Benford RD, Snow DA (2000). Framing processes and social movements: an overview and assessment. Annu Rev Sociol.

[54] Colombini M, Mayhew SH, Hawkins B (2016). Agenda setting and framing of gender-based violence in Nepal: how it became a health issue. Health Policy Plan.

[55] Parkhurst JO, Vulimiri M (2013). Cervical cancer and the global health agenda: insights from multiple policy-analysis frameworks. Glob Public Health.

[56] Mintrom M, Norman P (2009). Policy entrepreneurship and policy change. Policy Stud J.

[57] Bowen GA (2009). Document analysis as a qualitative research method. Qual Res J.

