# Supplementary material for: Why Was the Policy Idea on the Health Benefits Package Advisory Panel Gazetted in Kenya? A Retrospective Policy Analysis
Source: Int J Health Policy Manag. 2024 Jul 8;13:7608. doi: 10.34172/ijhpm.7608 (PMC11365168; doi:10.34172/ijhpm.7608)
Supplement: Supplementary file 1 — contains Tables S1-S3. [file ijhpm-13-7608-s001.pdf]

Article title: Why Was the Policy Idea on the Health Benefits Package Advisory Panel Gazetted in Kenya? A Retrospective Policy Analysis

Journal name: International Journal of Health Policy and Management (IJHPM)

Authors' information: Rahab Mbau[1,2]*, Anna Vassall[1], Lucy Gilson[1,3], Edwine Barasa[2,4,5]

[1]Department of Global Health and Development, London School of Hygiene and Tropical Medicine, London, UK.

[2]Health Economics Research Unit, KEMRI Wellcome Trust Research Programme, Nairobi, Kenya.

[3]School of Public Health and Family Medicine, University of Cape Town, Cape Town, South Africa.

[4]Centre for Global Health and Tropical Medicine, Nuffield Department of Medicine, University of Oxford, Oxford, UK.

[5]Institute of Healthcare Management, Strathmore University, Nairobi, Kenya.

*Correspondence to: Rahab Mbau; Email: waithirambau@gmail.com

Citation: Mbau R, Vassall A, Gilson L, Barasa E. Why was the policy idea on the Health Benefits Package Advisory Panel gazetted in Kenya? A retrospective policy analysis. Int J Health Policy Manag. 2024;13:7608. doi:10.34172/ijhpm.7608

Supplementary file 1

Table S1: Documents reviewed in the study

Types of documents	Examples (in electronic format)
Government documents (national policy documents, laws, and bills)	• 1st, 2nd, and 3rd Drafts of the Kenya Health Financing Strategy 2015-2030<br>• Second Medium-Term Plan 2013-2017 Transforming Kenya: Pathway to devolution, socio-economic development, equity, and national unity<br>• Sessional Paper No 2 on National Social Health Insurance in Kenya<br>• Health Act 2017<br>• Kenya Health Policy 2014-2030<br>• Cabinet Memorandum 2018- Roadmap to attain Universal Health Coverage in Kenya<br>• Gazette notices<br>• Report on the Stakeholder analysis to support design and finalization of the Health Financing Strategy for Kenya
Semi-autonomous Government Agencies' documents	• Report on National Health Insurance Fund Strategic Review and Market Assessment of Prepaid Health Schemes
Local research organizations' documents	• Published empirical studies, policy briefs, and reports
Campaign manifesto	• Transforming Kenya- Securing Kenya's prosperity 2013-2017<br>• Jubilee Manifesto 2017- Continuing Kenya's Transformation together
Development Partner reports and presentations	• Report on stakeholder analysis to support design and finalization of health financing strategy for Kenya<br>• World Health Organization meeting- Towards UHC in Kenya: issues, options, and guiding principles for the way forward<br>• World Bank Report on Moving towards UHC in Kenya
Media reports	• News media e.g., Online newspaper reports<br>• Web media e.g., Ministry of Health web portals and Development Partners web portals<br>• Social media e.g., Twitter

Table S2: Priority-setting bodies that develop benefits packages for Kenya's public health sector [39-41]

Priority-setting body	Type of benefits package	Purchaser
Directorate of policy and planning	Kenya Essential Package for Health	MOH & County Departments of Health
National Medicines and Therapeutics Committee	Kenya Essential Medicines List	MOH & County Departments of Health
Division of Vaccines and Immunization	Kenya Expanded Programme on Immunization	MOH & County Departments of Health

NHIF	• National Scheme benefit package<br>• Civil Servants and other Enhanced medical scheme benefit package<br>• Health Insurance Subsidy Program for the poor, elderly, and people with severe disabilities<br>• Health Insurance Subsidy Program for orphans and vulnerable children<br>• Free maternity Program<br>• Insurance program for secondary schools	NHIF
County Departments of Health	County specific health benefit packages	County Departments of Health

Table S3: Comparison of policy ideas [40,41]

**Priority-setting body responsible for designing the essential health benefits package**	**Pros**	**Cons**	**Implications**
MOH	a) Assures harmonization and standardization of benefits	a) Difficulty in managing stakeholders' interests<br>b) bureaucracies	a) necessitates legislation and regulation<br>b) necessitates governance and operations structures
Independent expert body or a health benefits expert committee (Advisory body composed of epidemiologists, health economists, health professionals, statisticians, health insurers, consumer organizations and experts in health technology assessment)	a) less bureaucracies<br>b) faster stakeholders buy-in<br>c) dedicated and committed team<br>d) standardization of the package across different purchasers	a) administrative and operational costs	a) necessitates legislation and regulation
Other Purchasers	a) understand the market risk<br>b) less bureaucracy in decision-making<br>c) may enhance creativity and innovation due to competition	a) conflict of interest<br>b) enforcing regulation will be difficult	a) necessitates regulation and legislation
